# Independent and Joint Associations of BMI and Waist Circumference With the Onset of Type 2 Diabetes Mellitus in Chinese Adults: Prospective Data Linkage Study

**DOI:** 10.2196/39459

**Published:** 2023-01-11

**Authors:** Sixuan Li, Yong Wang, Yanyan Ying, Qinghai Gong, Ge Lou, Yang Liu, Shiwei Liu, Hui Li

**Affiliations:** 1 Ningbo Municipal Center for Disease Control and Prevention Ningbo China; 2 Center for Disease Control and Prevention of Chaoyang District Beijing China; 3 Shanghai Pudong New Area Center for Disease Control and Prevention Pudong Institute of Preventive Medicine Fudan University Shanghai China; 4 Chinese Center for Disease Control and Prevention Bejing China

**Keywords:** body mass index, waist circumference, type 2 diabetes mellitus, prospective study, data linkage, epidemiology, hazard ratio

## Abstract

**Background:**

General obesity and abdominal obesity, typically measured by BMI and waist circumference (WC), respectively, are associated with an increased risk of type 2 diabetes mellitus (T2DM). However, the magnitude of the association of these two obesity indicators and their joint association with the onset of T2DM remain controversial.

**Objective:**

The aim of this study was to investigate the associations between these two obesity indicators and T2DM among the Chinese population to contribute scientific evidence for appropriate T2DM interventions.

**Methods:**

A cohort of 3001 eligible participants was selected from the Ningbo Adult Chronic Disease Surveillance Project running since 2015. Based on BMI, individuals were categorized into groups of underweight or normal, overweight, and obesity. Based on WC, individuals were categorized in groups of normal, precentral obesity, and central obesity. Follow-up was performed by linking data of the baseline data set with the diabetes registry data set and the vital registry data set (both from the Ningbo Municipal Integrated Noncommunicable Disease Collaborative Management System), mainly using the participants’ identity numbers. Follow-up was completed when a participant was diagnosed with T2DM. The associations were estimated with multivariate Cox proportional hazard regression.

**Results:**

In the cohort, 90 of 3001 participants developed T2DM (incidence density: 6.483/1000 person-years) with a median 4.72 years of follow-up. After controlling for age, sex, hypertension, dyslipidemia, smoking status, and family history of diabetes, the multivariate adjusted hazard ratios (HRs) across underweight/normal, overweight, and obesity BMI categories were 1.000, 1.653 (95% CI 1.030-2.654), and 2.375 (95% CI 1.261-4.473), respectively. The multivariate adjusted HRs across the normal, precentral obesity, and central obesity WC categories were 1.000, 1.215 (95% CI 0.689-2.142), and 1.663 (95% CI 1.016-2.723), respectively. Compared with the reference group (normal WC with an underweight/normal BMI), the multivariate adjusted HR for participants with both central obesity according to WC and obesity according to BMI was 2.489 (95% CI 1.284-4.825).

**Conclusions:**

Both elevated BMI and WC at baseline increased the risk of T2DM. Compared with WC, BMI as an obesity indicator was more strongly associated with the onset of T2DM.

## Introduction

Diabetes mellitus is a group of metabolic disorders characterized by elevated blood glucose levels, which can lead to various health problems and severe complications with disease progression [1]. A nationwide survey in 2015-2017 reported that the prevalence of diabetes was 12.8% in China [2]. Obesity is as an important modifiable risk factor for type 2 diabetes mellitus (T2DM), and the prevalence of overweight, obesity, and abdominal obesity among the Chinese population was estimated at 28.1%, 5.2%, and 29.1%, respectively, in 2012-2015 [3,4].

BMI and waist circumference (WC) are two of the key indicators used to measure general obesity and abdominal obesity, respectively. Some studies found that compared with that of people without T2DM, the proportion of body fat distributed in the abdominal area was higher among patients who were not classified as obese according to BMI criteria, whereas some people with obesity were metabolically healthy [5,6]. Since BMI does not take the exact fat mass into account, especially the visceral adipose tissue, this indicator has a limitation in reflecting the body fat distribution [7]. It has been reported that WC might better reflect visceral obesity than BMI [8]. Recently, a growing body of studies indicated that WC was strongly associated with T2DM and is a better predictor for the onset of T2DM than BMI [9-11].

Although both general obesity and abdominal obesity have been confirmed to be associated with the risk of T2DM, the magnitude of these two obesity indicators and their joint association with T2DM remain controversial [12,13]. Moreover, the number of prospective studies addressing this issue is limited for the Chinese population. Therefore, we performed this prospective data-linkage study to investigate the independent and joint associations of these two obesity indicators with the risk of T2DM to provide better scientific evidence for T2DM interventions in Ningbo, China.

## Methods

### Study Design and Participants

#### Baseline Survey

We conducted the baseline survey (the Ningbo Adult Chronic Disease Surveillance Project) on October 26 and December 31, 2015. This was a population-based project with the aims of understanding the prevalence, awareness, treatment, and control situation, and to explore the relevant behavior risk factors of major chronic diseases (hypertension, diabetes mellitus, and dyslipidemia) among Chinese adult residents aged 15-79 years in Ningbo city.

A total of 5280 residents were selected using a multistage cluster random sampling method covering 11 districts and counties in Ningbo. In the first stage, three towns or streets were selected from each county or district using a proportionate-to-population size sampling scheme. In the second stage, two administrative villages or neighborhood communities were selected from each town or street using the same method as used in the first stage. In the third stage, one village containing at least 105 households was selected from each neighborhood community or administrative village. In the final stage, 105 randomly selected households from each village and one resident aged 15-74 years in each household whose date of birth was closest to the 15th was selected as the survey participant. Finally, a total of 5160 residents completed the survey with a response rate of 97.73% (5160/5280).

#### Inclusion and Exclusion Criteria for Baseline Participants

Inclusion criteria were as follows: (1) aged ≥40 years, (2) living in the current district or county for at least 6 months, and (3) capable of communicating in Mandarin or local dialects with reading skills. The exclusion criterion was having been diagnosed with diabetes by clinicians in hospitals or community health service centers at the time of the baseline survey. A total of 3001 eligible participants aged above 40 years were selected from the pool of 5160 participants. All participants signed the written informed consent form.

### Ethics Approval

The research protocol was approved by the ethics committee of Ningbo Center for Disease Control and Prevention (approval number 201702).

### Data Collection

The data collection methods included a questionnaire survey, physical measurements, and laboratory tests. The questionnaire consisted of five components, including physical activities, environment and facilities, signs and symptoms, dietary and drinking habits, and demographic characteristics. All questionnaires were filled in face-to-face by trained staff. The completeness and accuracy of the questionnaire were double-checked by quality control personnel following the quality control requirements. We measured the height, weight, WC, blood pressure, fasting plasma glucose (FPG), glycated hemoglobin (HbA_1c_), total cholesterol (TC), triglycerides (TG), high-density lipoprotein cholesterol (HDL-C), and low-density lipoprotein cholesterol (LDL-C) for all participants. The physical examinations were carried out by the investigators using uniform devices and methods. Participants fasted the night before measurements were taken. The height, weight, and WC were measured while the participant was wearing light clothing (shorts and T-shirt) and without shoes. The participants were required to avoid vigorous exercises, eating, and drinking beverages (especially caffeinated drinks) within 30 minutes before measuring blood pressure and to rest quietly for 5 minutes before the first measurement. The blood pressure was tested three times and the average was taken. Five milliliters of the fasting venous blood was collected from the participants to detect FPG, HbA_1c_, TC, TG, HDL-C, and LDL-C.

### Follow-up by Data Linkage

**Figure 1:**
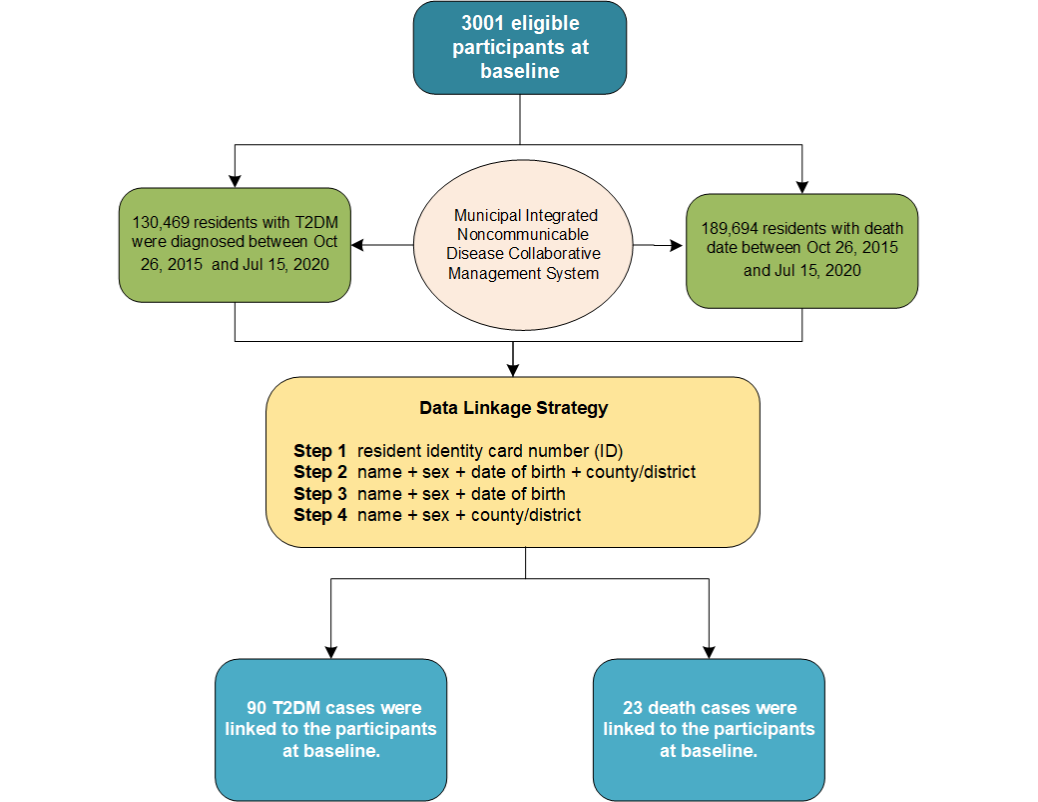
Flowchart of data linkage. T2DM: type 2 diabetes mellitus.

The data sources for the follow-up were obtained from the Ningbo Municipal Integrated Noncommunicable Disease Collaborative Management System (NCDCMS), which has continuously collected surveillance data since 2009 and covered the whole population of Ningbo. This system, which has been proven to obtain high-quality data and to have a low rate of underreporting (<5%), collects four types of noncommunicable disease data (diabetes mellitus, ischemic heart disease and cardiac arrest, cerebrovascular disease, malignant neoplasms) and cause of death data in a real-time manner [14]. We obtained the data from the diabetes registry with a diagnosis date from October 26, 2015, to July 15, 2020; the data of vital registration were collected according to a death date from October 26, 2015, to July 15, 2020. A total of 130,469 cases of T2DM and 189,694 cases of death were obtained from NCDCMS. The follow-up was completed by linking data of the baseline data set with the diabetes registry data set and the vital registration data set using the personal identification (ID) number. The participants who failed to link with these two data sets by ID number were linked via three combinations of identifiers in turn (ie, name+sex+date of birth+county/district; name+sex+date of birth; name+sex+county/district). A double check was performed to confirm whether the participants who successfully linked with two data sets via three identifiers were correct. Finally, 90 cases of T2DM and 23 deaths were linked to the participants at baseline through the data-linkage procedure. The flowchart of data linkage is provided in Figure 1.

### Definition of Outcome (Endpoint of Follow-up)

The endpoint of follow-up was a diagnosis of T2DM. The participants who failed to be linked with the diabetes registry data set were regarded as censored. According to the Chinese Type 2 Diabetes Prevention and Control 2017 Guidelines, T2DM was defined as typical clinical manifestations (thirst, frequent urination, overeating and unexplained weight loss), and (1) random plasma glucose≥11.1 mmol/L (200 mg/dL), (2) FPG≥7.0 mmol/L (126 mg/dL), or (3) oral glucose tolerance test 2-hour plasma glucose≥11.1 mmol/L (200 mg/dL) [15].

### Follow-up Time Calculation

The follow-up time was calculated as the date of diagnosis minus the start date of the baseline survey (October 26, 2015) for participants who linked with the diabetes registry data set, as the date of death minus the start date of the baseline survey (October 26, 2015) for participants who linked with the vital registration data set, and as the set end time of follow-up (July 15, 2020) minus the start date of the baseline survey (October 26, 2015) for participants who failed to link with these two data sets.

### Exposure

According to the 2013 Health Industry Standards of the People’s Republic of China “Criteria of Weight for Adults” and the World Health Organization report of WC and waist-to-hip ratio (WHR), BMI was categorized as underweight or normal (≤23.9 kg/m^2^), overweight (24-27.9 kg/m^2^), and obesity (≥28 kg/m^2^). WC was divided into three groups as follows: (1) normal: WC<85 cm for men and WC<80 cm for women; (2) precentral obesity: WC of 85-89.9 cm for men, WC of 80-84.9 cm for women; and (3) central obesity: WC≥90 cm for men WC≥85 cm for women [16,17].

### Covariates

#### Demographic and Lifestyle Factors

Demographic factors included age, sex, education level, marital status, residence place, and occupation. Lifestyle factors included smoking status, alcohol consumption, and physical activity level. Alcohol consumption was derived from the question: “Have you had any kind of alcoholic drink in the past 12 months?” Participants were divided into three groups: No; Yes, within 30 days; Yes, 30 days ago. Participants who currently smoke cigarettes or quit smoking were defined as ever smokers. Participants who never smoke cigarettes were defined as never smokers. Physical activity was classified into the following three levels in strict accordance with the Chinese guidelines for data processing and analysis of the International Physical Activity Questionnaire: low, moderate, and high. A moderate physical level was defined as (1) 3 or more days of vigorous-intensity activity of at least 20 minutes per day; (2) 5 or more days of moderate-intensity activity and/or walking at least 30 minutes per day; or (3) 5 or more days of any combination of walking, moderate-intensity activities, or vigorous-intensity activities achieving a minimum total physical activity of at least 600 metabolic equivalent (MET)-minutes/week. A high physical activity level was defined as: (1) vigorous-intensity activity at least 3 days per week achieving a minimum total physical activity of at least 1500 MET-minutes/week or (2) 7 or more days of any combination of walking, moderate-intensity activities, or vigorous-intensity activities achieving a minimum total physical activity of at least 3000 MET-minutes/week [18].

#### Medical Conditions

Hypertension was defined as systolic blood pressure≥140 mmHg, diastolic blood pressure≥90 mmHg, current use of antihypertensive medication, or self-reported history of hypertension [19]. Dyslipidemia was defined as TC≥5.2 mmol/L, TG≥1.7 mmol/L, HDL-C≤1.0 mmol/L, LDL-C≥3.4 mmol/L, current use of lipid-lowering medication, or self-reported history of dyslipidemia.

### Statistical Analyses

The incidence density of T2DM was obtained by dividing the number of T2DM cases by the person-years of follow-up. Normally distributed continuous data are described as mean (SD) and skewed-distributed continuous data are described as median (IQR). Categorical data are described as count (percentage). Differences in the distribution of baseline characteristics between participants with and without T2DM were evaluated using the Mann-Whitney *U* test for continuous variables and with the Pearson *χ*^2^ test for categorical variables. The strength of the association was measured using Cramer V for the *χ*^2^ test. Tests for linear trend across categories of BMI and WC were performed by treating the categories as a continuous variable with the median value for each category.

Univariate Cox regressions were performed for each exposure (BMI categories, central obesity categories) and covariates (residence place, sex, age, education level, marital status, occupation, family history of diabetes, alcohol consumption, smoking status, physical activity, hypertension, and dyslipidemia) to calculate the unadjusted hazard ratios (HRs) with 95% CIs. Age and sex–adjusted HRs with 95% CIs were estimated with Cox proportional hazard models stratified by BMI and central obesity categories adjusting for age and sex. Multivariate Cox proportional hazard models were performed to calculate HRs with 95% CIs based on the different categories of BMI and central obesity by adjusting for potential confounders (age, sex, hypertension, dyslipidemia, smoking status, family history of diabetes).

To estimate the joint effect of general obesity according to BMI and central obesity according to WC on the risk of T2DM, the participants were classified into six groups based on the BMI and WC categories. The adjusted HRs along with potential confounders were estimated using Cox models. BMI and WC were also included in the Cox regression model as continuous variables, either separately or together. The proportionality assumption of the hazards in the Cox regression analyses was tested by Schoenfeld residuals and the assumption was satisfied for all analyses. Forest plots were used to compare the independent and joint associations of BMI and WC. All data management and statistical analyses were performed using the statistical software R version 3.4.3. *P*<.05 was considered statistically significant.

## Results

### Baseline Characteristics of the Participants

The final cohort included 3001 participants at baseline with a median age of 54 years. These participants were followed up for a median of 4.72 years, comprising 13,880.8 person-years of follow-up. Overall, 90 of the 3001 participants developed T2DM, with an incidence density of 6.483 per 1000 person-years by the end of follow-up. As shown in Table 1, the participants who developed T2DM were older with higher baseline BMI, WC, FBG, and HbA_1c_, and with a greater proportion reporting a family history of diabetes compared with those who did not develop T2DM during the study period.

At baseline, 1570 (52.32%) of the participants were underweight or normal, 1159 (38.62%) were overweight, and 272 (9.06%) had obesity. Compared to the underweight or normal group, participants in the overweight and obesity groups tended to have higher WC, SBP, DBP, HbA_1c_, LDL-C, TC, and TG; lower HDL-C; and a higher proportion of hypertension and dyslipidemia. There were 1397 (46.55%) participants with normal WC, 721 (24.03%) with precentral obesity, and 865 (28.82%) with central obesity, demonstrating similar patterns as observed for BMI categories.

**Table 1:** Baseline characteristics of the 3001 participants by diabetes status.

Variables		Total (N=3001)	T2DM^a^ (n=90)	No T2DM (n=2911)	*P* value^b^	Cramer V
**Residence place, n (%)**					.50	0.01
	Urban	1573 (52.4)	44 (48.9)	1529 (52.5)		
	Rural	1428 (47.6)	46 (51.1)	1382 (47.5)		
**Sex, n (%)**					.56	0.009
	Male	1277 (42.6)	41 (45.6)	1236 (42.5)		
	Female	1724 (57.4)	49 (54.4)	1675 (57.5)		
**Education level, n (%)**					.37	0.026
	Primary school and above	1423 (47.4)	44 (48.9)	1379 (47.4)		
	Middle school	1048 (34.9)	35 (38.9)	1013 (34.8)		
	High school and above	530 (17.7)	11 (12.2)	519 (17.8)		
**Marital status, n (%)**					.52	0.028
	Single	46 (1.5)	2 (2.2)	44 (1.5)		
	Married	2734 (91.1)	78 (86.7)	2656 (91.2)		
	Widowed	151 (5.0)	7 (7.8)	144 (4.9)		
	Divorced/separated/ cohabitated	70 (2.3)	3 (3.3)	67 (2.3)		
**Occupation, n (%)**					.20	0.039
	Agriculture/husbandry/transportation	803 (26.8)	24 (26.7)	779 (26.8)		
	Government agency/enterprises	324 (10.8)	6 (6.7)	318 (10.9)		
	Other industries	589 (19.6)	13 (14.4)	576 (19.8)		
	Unemployed/housework/retired	1285 (42.8)	47 (52.2)	1238 (42.5)		
**Family history of diabetes, n (%)**					<.001	0.074
	No	2347 (78.2)	57 (63.3)	2290 (78.7)		
	Yes	407 (13.6)	25 (27.8)	382 (13.1)		
	I don’t know	247 (8.2)	8 (8.9)	239 (8.2)		
**Alcohol consumption, n (%)**					.67	0.016
	No	2062 (68.7)	62 (68.9)	2000 (68.7)		
	Yes, within 30 days	780 (26.0)	25 (27.8)	755 (25.9)		
	Yes, 30 days ago	159 (5.3)	3 (3.3)	156 (5.4)		
**Smoking status, n (%)**					.38	0.013
	Never smoker	2158 (71.9)	61 (67.8)	2097 (72.0)		
	Ever smoker	843 (28.1)	29 (32.2)	814 (28.0)		
**Physical activity, n (%)**					.53	0.021
	Low level	418 (13.9)	16 (17.8)	402 (13.8)		
	Median level	2455 (81.8)	71 (78.9)	2384 (81.9)		
	High level	128 (4.3)	3 (3.3)	125 (4.3)		
**Hypertension, n (%)**					<.001	0.079
	No	1492 (49.7)	24 (26.7)	1468 (50.4)		
	Yes	1509 (50.3)	66 (73.3)	1443 (49.6)		
**Dyslipidemia, n (%)**					.01	0.045
	No	1231 (41.0)	25 (27.8)	1206 (41.4)		
	Yes	1770 (59.0)	65 (72.2)	1705 (58.6)		
**BMI categories, n (%)**					<.001	0.072
	Underweight or normal	1570 (52.3)	30 (33.3)	1540 (52.9)		
	Overweight	1159 (38.6)	45 (50.0)	1114 (38.3)		
	Obesity	272 (9.1)	15 (16.7)	257 (8.8)		
**WC^c^ categories, n (%)**					.002	0.064
	Normal	1415 (47.2)	29 (32.2)	1386 (47.6)		
	Precentral obesity	721 (24.0)	21 (23.3)	700 (24.0)		
	Central obesity	865 (28.8)	40 (44.4)	825 (28.3)		
BMI (kg/m^2^), median (IQR)		23.81 (21.94-25.89)	25.14 (23.12-26.95)	23.77 (21.90-25.84)	<.001	N/A^d^
WC (cm), median (IQR)		82.25 (76.50-88.25)	86.00 (80.30-90.84)	82.20 (76.50-88.10)	<.001	N/A
FBG^e^ (mmol/L), median (IQR)		4.94 (4.60-5.38)	5.90 (5.23-6.99)	4.93 (4.59-5.36)	<.001	N/A
HbA_1c_^f^ (%), median (IQR)		4.70 (4.30-5.10)	5.40 (4.80-6.18)	4.70 (4.30-5.10)	<.001	N/A
Age (years), median (IQR)		54.00 (48.00-63.00)	58.00 (52.00-65.50)	54.00 (48.00-63.00)	<.001	N/A

^a^T2DM: type 2 diabetes mellitus.

^b^Differences in the distribution of participants with and without T2DM were evaluated using the Mann-Whitney *U* test for continuous variables and with the Pearson *χ*^2^ test for categorical variables.

^c^WC: waist circumference.

^d^N/A: not applicable.

^e^FBG: fasting blood glucose.

^f^HbA_1c_: glycated hemoglobin.

### Univariate Cox Regression Analysis

BMI categories, WC categories, age, occupation, family history of diabetes, hypertension, and dyslipidemia were significantly associated with the incidence of T2DM. Compared with participants of normal weight, the overweight (HR 2.052, 95% CI 1.293-3.258) and obesity (HR 2.924, 95% CI 1.573-5.434) categories were significantly associated with an increased risk of the development of T2DM. There was a dose-response relationship between the BMI categories and the onset of T2DM (*P* for trend <.001). Compared with participants in the normal WC group as reference, central obesity (HR 2.289, 95% CI 1.419-3.691) was significantly associated with an increased risk of the development of T2DM (*P* for trend <.001). The risk of T2DM onset was significantly increased with the increase of age. The HRs for age groups of 40-49 years, 50-59 years, 60-69 years, and ≥70 years were 1.000, 3.670, 3.423, and 5.279, respectively. The risk of T2DM in participants with hypertension and dyslipidemia was respectively 2.754 (95% CI 1.726-4.393) and 1.823 (95% CI 1.149-2.891) higher that of participants free of diseases.

### Independent and Joint Associations of BMI and WC on the Risk of T2DM

**Figure 2:**
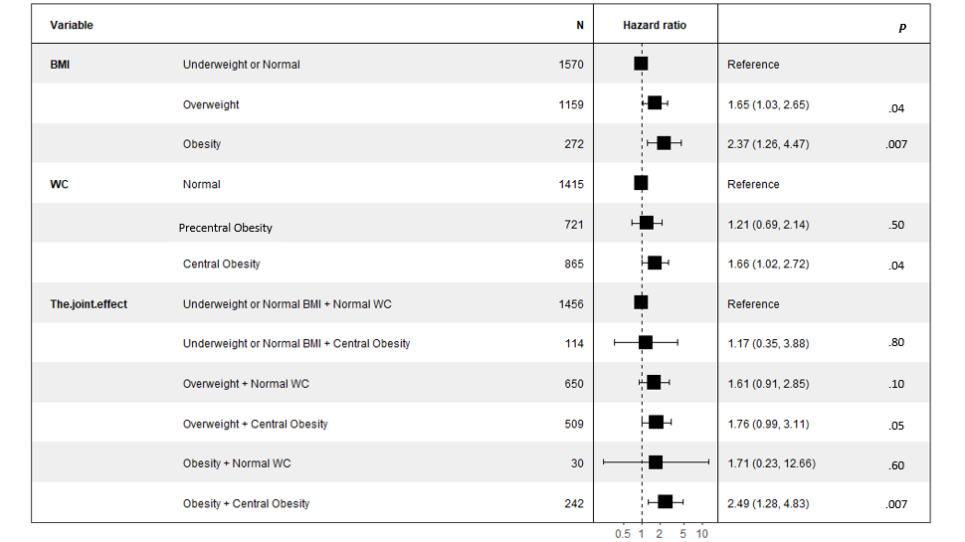
Forest plot of the association between categories of BMI and/or waist circumference (WC) and the onset of type 2 diabetes mellitus adjusting for age, gender, hypertension, dyslipidemia, smoking, and family history of diabetes.

The independent and joint associations of BMI and WC with T2DM development are displayed in Table 2 and Figure 2. The age and sex–adjusted HRs increased across categories of BMI and the same pattern was found after adjustment for potential confounders with a slight decrease, although the trend remained significant (*P* for trend <.001). The age and sex–adjusted HRs also increased across categories of WC (*P* for trend .003) and the trend remained significant after adjustment of potential confounders (*P* for trend .04).

Among the participants who were categorized in the central obesity group, the age and sex–adjusted HRs increased successively for those categorized in the underweight or normal, overweight, and obesity groups according to BMI compared with the reference group (normal WC with an underweight or normal BMI). Compared to the reference group, the multivariate adjusted HR for participants with both central obesity according to WC and obesity according to BMI was much higher than that for the independent association of central obesity or obesity (Table 2).

When BMI and WC were treated as continuous variables, the HR of BMI was still slightly higher than that of WC. When the BMI and WC were considered together in the regression model using mutual adjustment, BMI, but not WC, was significantly associated with the risk of T2DM (Table 3).

**Table 2:** Risk of type 2 diabetes mellitus stratified by categories of BMI and waist circumference (WC) in univariate, age and sex–adjusted, and multivariate-adjusted regressions.

Categories		Univariate, HR^a^ (95% CI)	Age and sex–adjusted^b^, HR (95% CI)	Multivariate^c^, HR (95% CI)
**BMI categories**				
	Underweight or normal (n=1570)	1	1	1
	Overweight (n=1159)	2.052 (1.293-3.258)	1.941 (1.221-3.087)	1.653 (1.030-2.654)
	Obesity (n=272）	2.924 (1.573-5.434)	2.918 (1.569-5.425)	2.375 (1.261-4.473)
	*P* for trend	<.001	<.001	.004
**WC categories**				
	Normal WC (n=1415)	1	1	1
	Precentral obesity (n=721)	1.425 (0.813-2.499)	1.374 (0.767-2.364)	1.215 (0.689-2.142)
	Central obesity (n=865)	2.289 (1.419-3.691)	2.059 (1.273-2.059)	1.663 (1.016-2.723)
	*P* for trend	<.001	.003	.04
**Joint effect of BMI and WC**				
	Underweight or normal BMI+normal WC (n=1456)	1	1	1
	Underweight or normal BMI+central obesity (n=114)	1.418 (0.430-4.676)	1.316 (0.398-4.352)	1.170 (0.353-3.878)
	Overweight+normal WC (n=650)	1.833 (1.044-3.218)	1.819 (1.035-3.198)	1.608 (0.908-2.847)
	Overweight+central obesity (n=509)	2.479 (1.421-4.323)	2.185 (1.249-3.823)	1.756 (0.991-3.110)
	Obesity+normal WC (n=30)	1.777 (0.241-13.07)	1.913 (0.259-14.08)	1.713 (0.231-12.65)
	Obesity+central obesity (n=242)	3.169 (1.662-6.044)	3.116 (1.633-5.945)	2.489 (1.284-4.825)

^a^HR: hazard ratio.

^b^Adjusted for age and sex.

^c^Adjusted for age, sex, hypertension, dyslipidemia, smoking, and family history of diabetes.

**Table 3:** Risk of type 2 diabetes based on BMI and waist circumference (WC) as continuous variables.

Models		Univariate, HR^a^ (95% CI)	Age and sex–adjusted^b^, HR (95% CI)	Multivariate^c^, HR (95% CI)
**Independent effects**				
	BMI	1.142 (1.078-1.210)	1.141 (1.076-1.210)	1.118 (1.049-1.191)
	WC	1.041 (1.019-1.063)	1.038 (1.015-1.061)	1.029 (1.004-1.054)
**Joint effects^d^**				
	BMI	1.116 (1.021-1.221)	1.143 (1.041-1.254)	1.130 (1.027-1.243)
	WC	1.011 (0.979-1.045)	1.000 (0.965-1.035)	0.995 (0.960-1.031)

^a^HR: hazard ratio.

^b^Adjusted for age and sex.

^c^Adjusted for age, sex, hypertension, dyslipidemia, smoking, and family history of diabetes.

^d^BMI and WC were considered together in the regression model.

## Discussion

### Principal Findings and Comparison to Previous Work

In this study, we found that both elevated BMI and WC at baseline increased the risk of T2DM. Moreover, the magnitude of association of BMI with T2DM was stronger than that of WC, regardless of treating the exposures as continuous or categorical variables, which was consistent with previous studies conducted in a Chinese population [20-22]. A prospective study performed over 7.26 years of follow-up in a southeast province of China indicated that BMI may be superior to WC in predicting the incidence of T2DM in the Chinese population [20]. A cohort study including 27,009 retirees in a central province concluded that BMI was the strongest predictor among various anthropometric indices with 4.6 years of follow-up data [22]. However, other studies reported inconsistent conclusions [23-26]. Kodama et al [23] reported that the pooled relative risk of T2DM per 1-SD increase in BMI was significantly lower than that for WC, indicating that WC was more strongly associated with diabetes risk than BMI. Lee et al [24] concluded that although WC had a stronger association with the risk of T2DM in comparison with BMI, there was no overall advantage for WC at discriminating the risk of developing T2DM compared with BMI.

The discrepancies in conclusions among studies were likely due to the differences in ethnicity of the populations. Since there is an assumption that the superiority of WC to BMI for T2DM prediction was based on the characteristics of the study population, previous studies mainly conducted in white-dominant populations or in the western sociocultural context might lead to discrepancies [23-25,27]. This study adds to this evidence for the Chinese population. Another reason for the inconsistent conclusions might be the length of follow-up. Aging is associated with progressive changes in the total and regional fat distribution [22]. As age increases, the body fat distribution changes with a decrease in lower subcutaneous fat and an increase in abdominal fat [28]. The results of previous studies might also be distorted by the increasing follow-up time as more participants tended to have abdominal obesity, which diluted the overall impact of baseline body fat distribution [23]. Differences in research methods and populations can also lead to different conclusions. Although a large-population prospective study in Chinese adults showed that WC was a better predictor than BMI for the onset of T2DM in women, this cohort study used a logistic regression model rather than a Cox regression model to predict the magnitude of associations [29]. The effect of time of follow-up was not considered in the model, which might have also affected the results. Moreover, as a cohort study carried out in an employee population, the “healthy worker effect” might have impacted these results [29]. Furthermore, the inconsistent conclusions might also be related to the different classification criteria of BMI and WC in different studies. Some studies obtained conclusions using categories of BMI or WC, whereas others used the SD.

In our study, after 4.72 years of follow-up, 90 participants developed T2DM with a total incidence density of 6.483 per 1000 person-years, similar to the study of Yang et al [21] (0.89/100 person-years). However, this incidence density was much lower than that reported in some previous longitudinal studies [30,31]. In a 10-year population-based Asian Indian cohort study, the incidence density of diabetes was 22.2 per 1000 person-years [32]. The cumulative incidence of T2DM was 14.4 and 13.7 per 1000 person-years for men and women, respectively, in an 18-year follow-up cohort study performed in Mexico [30]. The relatively low incidence density in our study may be attributed to the small number of participants at baseline, relatively young baseline population, and short follow-up time.

### Limitations and Future Work

There were some limitations of this study. First, the number of participants at baseline was relatively small and the follow-up time was short. Referring to the joint associations of BMI and WC adjusting for age and sex, the HRs revealed a dose-response relationship in the overweight category. However, due to the short time of follow-up and relatively young baseline participants in this study, only 90 participants were diagnosed with T2DM. The number of newly diagnosed T2DM participants in each BMI category might not have been sufficient to detect the dose-response relationship with the increase of WC. Second, the distribution and composition of body fat might gradually change during follow-up, along with the corresponding BMI and WC. In this study, the change values of BMI and WC during follow-up were not adjusted in the models owing to the study design, which may have influenced the results. Data linkage, the key technique used in this study, has become a popular research method for generating population-level electronic cohorts, and is used as a supplement to conventional cohort studies [14,29]. Data linkage provides a convenient and cost-effective approach for collecting data from multichannel sources and investigating the causes and development in the variation of outcomes across the individual’s life course [29,33]. Nevertheless, compared with a conventional cohort study, data linkage has some limitations and might introduce bias into results due to linkage errors (false matches or missed matches). Despite NCDCMS being confirmed as a platform with high data quality and a very low underreporting rate, some linkage errors are still inevitable [14]. In this study, we tried to avoid linkage errors by using three combinations of identifiers as a complement to the ID and double checking whether the cases linked via three combinations of identifiers were correct. Moreover, we controlled the proven and possible confounding factors in the models collected from the baseline survey and strictly controlled the quality of data during the investigation. Additionally, since the follow-up was completed by data linkage based on NCDCMS in Ningbo, the results might not be generalizable to other cities.

The Chinese population generally has a higher percentage of body fat and a greater propensity to central obesity compared with those of western counterparts under the same BMI [34]. Although BMI as an obesity indicator was more strongly associated with the risk of T2DM than WC in this study, we recommend that the general population and people at high risk of T2DM should focus on control of body weight as well as WC, which is consistent with the Chinese Type 2 Diabetes Prevention and Control Guideline [15]. In addition to obesity, the risk of developing T2DM was significantly higher in people who showed comorbid hypertension or dyslipidemia in comparison with participants who were free of diseases, indicating the importance of blood pressure control and maintaining a normal lipid level for the general population and especially for people at high risk of T2DM. In addition, a healthy lifestyle, including a balanced diet, regular physical activities, tobacco cessation, and alcohol control, is highly recommended, especially in the elderly population.

A study with longer-term follow-up is needed to further investigate the joint association of BMI and WC with the risk of T2DM. Although BMI, WC, and the WHR are inexpensive and easy measures to assess the adipose tissue, there is growing interest in using magnetic resonance techniques for evaluating total body fat and different fat compartments in western countries. Magnetic resonance imaging provides high accuracy in quantitatively detecting the subcutaneous and visceral adipose tissue, which could be an option to further explore the association between obesity and T2DM accurately [35]. Additionally, performing a meta-analysis for studies in the Chinese population is suggested to better detect the pooled relative risk by removing the influence of ethnicity.

### Conclusion

In conclusion, both elevated BMI and WC at baseline increased the risk of T2DM. Compared with WC, BMI as an obesity indicator was more strongly associated with the onset of T2DM among Chinese adults in Ningbo. We recommend that residents of this population, especially the elderly, maintain a normal weight and a healthy lifestyle.

## Abbreviations

FPG: fasting plasma glucose
HbA_1c_: glycated hemoglobin
HDL-C: high-density lipoprotein cholesterol
HR: hazard ratio
ID: identification
LDL-C: low-density lipoprotein cholesterol
MET: metabolic equivalent
NCDCMS: Ningbo Municipal Integrated Noncommunicable Disease Collaborative Management System
T2DM: type 2 diabetes mellitus
TC: total cholesterol
TG: triglycerides
WC: waist circumference
WHR: waist-to-hip ratio

## References

[1] Diabetes. World Health Organization.

[2] Li Y, Teng D, Shi X, Qin G, Qin Y, Quan H, Shi B, Sun H, Ba J, Chen B, Du J, He L, Lai X, Li Y, Chi H, Liao E, Liu C, Liu L, Tang X, Tong N, Wang G, Zhang JA, Wang Y, Xue Y, Yan L, Yang J, Yang L, Yao Y, Ye Z, Zhang Q, Zhang L, Zhu J, Zhu M, Ning G, Mu Y, Zhao J, Teng W, Shan Z (2020). Prevalence of diabetes recorded in mainland China using 2018 diagnostic criteria from the American Diabetes Association: national cross sectional study. BMJ.

[3] Zhang L, Wang Z, Wang X, Chen Z, Shao L, Tian Y, Dong Y, Zheng C, Li S, Zhu M, Gao R, China Hypertension Survey investigators (2019). Prevalence of abdominal obesity in China: results from a cross-sectional study of nearly half a million participants. Obesity.

[4] Zhang L, Wang Z, Wang X, Chen Z, Shao L, Tian Y, Zheng C, Li S, Zhu M, Gao R, China Hypertension Survey investigators (2020). Prevalence of overweight and obesity in China: results from a cross-sectional study of 441 thousand adults, 2012-2015. Obes Res Clin Pract.

[5] (2020). HEARTS-D: diagnosis and management of type 2 diabetes. World Health Organization.

[6] Wildman R, Muntner P, Reynolds K, McGinn A, Rajpathak S, Wylie-Rosett J, Sowers MFR (2008). The obese without cardiometabolic risk factor clustering and the normal weight with cardiometabolic risk factor clustering: prevalence and correlates of 2 phenotypes among the US population (NHANES 1999-2004). Arch Intern Med.

[7] Kim Y, Kim S, Han K, Jung J, Lee S, Oh S, Park H, Rhee E, Lee W, Yoo S (2019). Waist circumference and all-cause mortality independent of body mass index in Korean population from the National Health Insurance Health Checkup 2009⁻2015. J Clin Med.

[8] Nazare JA, Smith J, Borel AL, Aschner P, Barter P, Van Gaal L, Tan CE, Wittchen HU, Matsuzawa Y, Kadowaki T, Ross R, Brulle-Wohlhueter C, Alméras N, Haffner SM, Balkau B, Després JP, INSPIRE ME IAA Investigators (2015). Usefulness of measuring both body mass index and waist circumference for the estimation of visceral adiposity and related cardiometabolic risk profile (from the INSPIRE ME IAA Study). Am J Cardiol Internet.

[9] Rosenthal AD, Jin F, Shu X, Yang G, Elasy TA, Chow W, Ji B, Xu H, Li Q, Gao Y, Zheng W (2004). Body fat distribution and risk of diabetes among Chinese women. Int J Obes Relat Metab Disord.

[10] Hou X, Chen S, Hu G, Chen P, Wu J, Ma X, Yang Z, Yang W, Jia W, China National Diabetes‚ Metabolic Disorders Study Group (2019). Stronger associations of waist circumference and waist-to-height ratio with diabetes than BMI in Chinese adults. Diabetes Res Clin Pract.

[11] Wang Y, Rimm E, Stampfer M, Willett W, Hu F (2005). Comparison of abdominal adiposity and overall obesity in predicting risk of type 2 diabetes among men. Am J Clin Nutr.

[12] Fan Y, Wang R, Ding L, Meng Z, Zhang Q, Shen Y, Hu G, Liu M (2020). Waist circumference and its changes are more strongly associated with the risk of type 2 diabetes than body mass index and changes in body weight in Chinese adults. J Nutr.

[13] Dale CE, Fatemifar G, Palmer TM, White J, Prieto-Merino D, Zabaneh D, Engmann JE, Shah T, Wong A, Warren HR, McLachlan S, Trompet S, Moldovan M, Morris RW, Sofat R, Kumari M, Hyppönen E, Jefferis BJ, Gaunt TR, Ben-Shlomo Y, Zhou A, Gentry-Maharaj A, Ryan A, de Mutsert R, Noordam R, Caulfield MJ, Jukema JW, Worrall BB, Munroe PB, Menon U, Power C, Kuh D, Lawlor DA, Humphries SE, Mook-Kanamori DO, Sattar N, Kivimaki M, Price JF, Davey Smith G, Dudbridge F, Hingorani AD, Holmes MV, Casas JP, UCLEB Consortium; METASTROKE Consortium (2017). Causal associations of adiposity and body fat distribution with coronary heart disease, stroke subtypes, and type 2 diabetes mellitus: a Mendelian randomization analysis. Circulation.

[14] Li S, Zhang L, Liu S, Hubbard R, Li H (2020). Surveillance of noncommunicable disease epidemic through the integrated noncommunicable disease collaborative management system: feasibility pilot study conducted in the city of Ningbo, China. J Med Internet Res.

[15] Chinese Diabetes Society (2018). Guidelines for the prevention and control of type 2 diabetes in China (2017 Edition). Chinese J Pract Intern Med.

[16] World Health Organization (2011). Waist circumference and waist-hip ratio: Report of a WHO Expert Consultation.

[17] People's Republic of China National Health and Family Planning Commission (2013). WS/T 428-2013 Criteria of weight for adults. Chinese Standard.

[18] Fan M, Lyu J, He P (2014). Chinese guidelines for data processing and analysis concerning the International Physical Activity Questionnaire. Zhonghua Liu Xing Bing Xue Za Zhi.

[19] Chinese Hypertension League (2019). Guidelines for the prevention and control of hypertension in China (2018 revised Edition). Chinese J Cardiovasc Med.

[20] Wang H, Hu R, Qian Y, Wang C, Xie K (2018). Prospective study on the effect of BMI and waist circumference on diabetes of adults in Zhejiang province. Chin J Epidemiol.

[21] Yang X, Zhang M, Luo X, Jinjin W, Yin L (2016). Body mass index, waist circumference and waist-to-height ratio associated with the incidence of type 2 diabetes mellitus: a cohort study. Chin J Epidemiol.

[22] Yang J, Wang F, Wang J, Han X, Hu H, Yu C, Yuan J, Yao P, Miao X, Wei S, Wang Y, Chen W, Liang Y, Guo H, Zhang X, Zheng D, Tang Y, Yang H, He M (2018). Using different anthropometric indices to assess prediction ability of type 2 diabetes in elderly population: a 5 year prospective study. BMC Geriatr.

[23] Kodama S, Horikawa C, Fujihara K, Heianza Y, Hirasawa R, Yachi Y, Sugawara A, Tanaka S, Shimano H, Iida KT, Saito K, Sone H (2012). Comparisons of the strength of associations with future type 2 diabetes risk among anthropometric obesity indicators, including waist-to-height ratio: a meta-analysis. Am J Epidemiol.

[24] Lee CMY, Woodward M, Pandeya N, Adams R, Barrett-Connor E, Boyko EJ, Eliasson M, Franco LJ, Fujimoto WY, Gonzalez C, Howard BV, Jacobs DR, Keinanen-Kiukaanniemi S, Magliano D, Schreiner P, Shaw JE, Stevens J, Taylor A, Tuomilehto J, Wagenknecht L, Huxley RR, Obesity‚ DiabetesCardiovascular Disease Collaboration (2017). Comparison of relationships between four common anthropometric measures and incident diabetes. Diabetes Res Clin Pract.

[25] Taylor A, Ebrahim S, Ben-Shlomo Y, Martin R, Whincup P, Yarnell J, Wannamethee SG, Lawlor DA (2010). Comparison of the associations of body mass index and measures of central adiposity and fat mass with coronary heart disease, diabetes, and all-cause mortality: a study using data from 4 UK cohorts. Am J Clin Nutr.

[26] Jia Z, Zhou Y, Liu X, Wang Y, Zhao X, Wang Y, Liang W, Wu S (2011). Comparison of different anthropometric measures as predictors of diabetes incidence in a Chinese population. Diabetes Res Clin Pract.

[27] Gallagher D, Visser M, Sepúlveda D, Pierson RN, Harris T, Heymsfield SB (1996). How useful is body mass index for comparison of body fatness across age, sex, and ethnic groups?. Am J Epidemiol.

[28] Kuk JL, Saunders TJ, Davidson LE, Ross R (2009). Age-related changes in total and regional fat distribution. Ageing Res Rev.

[29] Jutte DP, Roos LL, Brownell MD (2011). Administrative record linkage as a tool for public health research. Annu Rev Public Health.

[30] González-Villalpando C, Dávila-Cervantes CA, Zamora-Macorra M, Trejo-Valdivia B, González-Villalpando ME (2014). Incidence of type 2 diabetes in Mexico: results of the Mexico City Diabetes Study after 18 years of follow-up. Salud Publica Mex.

[31] Man RE, Charumathi S, Gan ATL, Fenwick EK, Tey CS, Chua J, Wong T, Cheng C, Lamoureux EL (2017). Cumulative incidence and risk factors of prediabetes and type 2 diabetes in a Singaporean Malay cohort. Diabetes Res Clin Pract.

[32] Anjana R, Shanthi Rani CS, Deepa M, Pradeepa R, Sudha V, Divya Nair H, Lakshmipriya N, Subhashini S, Binu VS, Unnikrishnan R, Mohan V (2015). Incidence of diabetes and prediabetes and predictors of progression among Asian Indians: 10-year follow-up of the Chennai Urban Rural Epidemiology Study (CURES). Diabetes Care.

[33] Hyler SE, Skodol AE, Oldham JM, Kellman HD, Doidge N (1992). Validity of the Personality Diagnostic Questionnaire-Revised: a replication in an outpatient sample. Compr Psychiatry.

[34] WHO Expert Consultation (2004). Appropriate body-mass index for Asian populations and its implications for policy and intervention strategies. Lancet.

[35] Schwenzer NF, Machann J, Schraml C, Springer F, Ludescher B, Stefan N, Häring H, Fritsche A, Claussen CD, Schick F (2010). Quantitative analysis of adipose tissue in single transverse slices for estimation of volumes of relevant fat tissue compartments: a study in a large cohort of subjects at risk for type 2 diabetes by MRI with comparison to anthropometric data. Invest Radiol.

